# Neuropathology of multiple system atrophy: Kurt Jellinger`s legacy

**DOI:** 10.1007/s00702-021-02383-3

**Published:** 2021-07-28

**Authors:** Nicole Campese, Alessandra Fanciulli, Nadia Stefanova, Johannes Haybaeck, Stefan Kiechl, Gregor K. Wenning

**Affiliations:** 1 Neurology Unit, Department of Clinical and Experimental Medicine, University of Pisa, Via Roma 67, 56126 Pisa, Italy; 2 Department of Neurology, Medical University of Innsbruck, Anichstrasse 35, 6020 Innsbruck, Austria; 3 Institute of Pathology, Neuropathology and Molecular Pathology, Medical University of Innsbruck, Müllerstrasse 44, 6020 Innsbruck, Austria; 4 Diagnostic & Research Center for Molecular BioMedicine, Institute of Pathology, Medical University Graz, Neue Stiftingtalstrasse 6, 8010 Graz, Austria

**Keywords:** Glial cytoplasmic inclusions, Multiple system atrophy, Neuropathology, Olivopontocerebellar atrophy, Striatonigral degeneration

## Abstract

Multiple System Atrophy (MSA) is a rare, fatal neurodegenerative disorder. Its etiology and exact pathogenesis still remain poorly understood and currently no disease-modifying therapy is available to halt or slow down this detrimental neurodegenerative process. Hallmarks of the disease are α-synuclein rich glial cytoplasmic inclusions (GCIs). Neuropathologically, various degrees of striatonigral degeneration (SND) and olivopontocerebellar atrophy (OPCA) can be observed. Since the original descriptions of this multifaceted disorder, several steps forward have been made to clarify its neuropathological hallmarks and key pathophysiological mechanisms. The Austrian neuropathologist Kurt Jellinger substantially contributed to the understanding of the underlying neuropathology of this disease, to its standardized assessment and to a broad systematical clinic-pathological correlation. On the occasion of his 90th birthday, we reviewed the current state of the art in the field of MSA neuropathology, highlighting Prof. Jellinger’s substantial contribution.

## Introduction

Multiple System Atrophy (MSA) is a rare, fatal, adult-onset neurodegenerative disorder with a mean incidence of 0.6–0.7 cases per 100000 person-years (Fanciulli and Wenning 2015). MSA is clinically characterized by early progressive autonomic failure (Fanciulli et al. 2019a) combined with motor impairment presenting with poorly L-dopa responsive parkinsonism, cerebellar features or a combination of both (Gilman et al. 2008). To date, a definite MSA diagnosis relies on neuropathological examination (Gilman et al. 2008). Based on the predominant motor phenotype, two main variants can be identified: the parkinsonian variant (MSA-p) and the cerebellar variant (MSA-c). These two clinical phenotypes correlate with the neuropathological patterns of predominant striatonigral degeneration (SND) and olivopontocerebellar atrophy (OPCA), respectively (Jellinger 2014a). In the western hemisphere, the parkinsonian variant outnumbers the cerebellar one (Köllensperger et al. 2010), while in Japan MSA-c occurs more frequently than MSA-p (Watanabe et al. 2002). Males and females are equally affected (Fanciulli and Wenning 2015) with symptoms’ onset occurring usually in the 6^th^ decade; nonetheless both young-onset (YOMSA) and late-onset variants, with disease onset before 40 years old and after 75 years old respectively, have been described (Batla et al. 2018; Fanciulli et al. 2019b). The mean survival is approximately 9.8 years (Wenning et al. 2013; Low et al. 2015), but both cases of prolonged survival (Petrovic et al. 2012) and of very rapidly progressive disease course with an overall survival of less than 3 years (Wakabayashi et al. 2005) have been reported.

No disease-modifying therapies are up to date available to halt or slow down the neurodegenerative process, representing an urgent, but still unmet need in MSA (Meissner et al. 2019). A better understanding of the underlying neuropathological changes is pivotal for identifying new therapeutic targets, developing animal models better reproducing the human pathology and the discovery and validation of diagnostic and prognostic markers opening new scenarios for interventional trials (Heras-Garvin and Stefanova 2020a). The Austrian neuropathologist Kurt Jellinger substantially contributed to our current knowledge on the neuropathological features of MSA. Here we provide an overview of the state of the art in the field of neuropathology in MSA, highlighting Jellinger’s role in dissecting “the nature of the beast” (Quinn 1989).

## Neuropathology

MSA belongs to the broad spectrum of α-synucleinopathies, a group of neurodegenerative disorders characterized by the abnormal accumulation of misfolded hyperphosphorylated α-synuclein (Jellinger 2003, 2019a). Pathological hallmarks of the disease are widespread oligodendroglial cytoplasmic inclusions (GCIs or Papp-Lantos bodies) (Papp et al. 1989; Papp and Lantos 1994), in which insoluble α-synuclein aggregates are detected (Tu et al. 1998) and whose distribution differs in the two main clinical phenotypes. Depending on the predominant parkinsonian or cerebellar features, various degrees of striatonigral (SND) and olivopontocerebellar degeneration (OPCA) can be observed in MSA.

### Macroscopic findings

Severe atrophy of the cerebellum, the middle cerebellar peduncle and the pontine nuclei associated with mild diffuse cortical atrophy in the frontal lobes are observed at the macroscopical examination of MSA brains (Jellinger 2014a, 2018a). By slicing the brain, gray discoloration of the posterolateral putamen often accompanied by pallor in the locus coeruleus (LC) and in the substantia nigra (SN) is detectable in MSA-p. Brown discoloration of the cerebellar white matter, narrowing of the cerebellar folia, various degrees of degeneration of paleo- and neocerebellum accompanied by atrophy of the pontine basis and of the middle cerebellar peduncle are core features of MSA-c (Jellinger 2018a).

### Histopathology

The following main histopathological features characterize MSA: α-synuclein-immunoreactive cellular inclusions, selective neuronal loss and axonal degeneration, myelin pallor with degeneration, microglial activation and astrogliosis (Trojanowski and Revesz 2007; Jellinger 2019a; Heras-Garvin and Stefanova 2020a).

A positive correlation between GCIs load and the severity of neuronal loss has been observed, suggesting a pivotal role of Papp-Lantos bodies or at least their components in driving the neurodegenerative process (Ozawa et al. 2004). Myelin degeneration with pallor and reduction in myelin basic protein (MBP) are widespread but more pronounced in the putamen and middle cerebellar peduncle, where they can be detected in vivo by diffusion tensor imaging (DTI) MRI techniques (Brooks and Seppi 2009). Microglial activation and astrogliosis, reflecting both the α-synuclein-pathology burden and neuronal loss are more pronounced in degenerating white matter areas showing mild to moderate myelin loss (Ishizawa et al. 2008).

### Inclusion pathology

α-synuclein-immunoreactive cellular inclusions, detectable by means of silver staining or anti-synuclein-antibody-based immunostainings, represent a pathognomonic feature of MSA. Besides GCIs, glial nuclear inclusions (GNIs), neuronal cytoplasmic inclusions (NCIs) and the neuronal nuclear inclusions (NNIs) can be observed in histological sections (Kaji et al. 2020).

According to the 2008 recommendations for the post-mortem neuropathological diagnosis of MSA “a definite neuropathological diagnosis of MSA is established when there is evidence of widespread and abundant CNS alpha-synuclein-positive GCIs in association with neurodegenerative changes in striatonigral or olivopontocerebellar structures” (Trojanowski and Revesz 2007).

GCIs are non-membrane-coated argyrophilic, triangle, half-moon- or sickle-shaped cytoplasmic aggregates detected in glial cells (Papp et al. 1989; Jellinger 2014a). Ultrastructurally Ser129-phosphorylated α-syn fibrils represent the main component of GCIs (Spillantini et al. 1998). α- and ß-tubulin, ubiquitin, 14–3-3 protein and fragments of cellular organelles (e.g., mitochondria, secretory vesicles) are also detected (Arima et al. 1992; Burn and Jaros 2001; McCormack et al. 2016). These components can also be found in Lewy Bodies (LB), but their proportion and ultrastructural features differ in these inclusion bodies (McCormack et al. 2016; Kaji et al. 2020).

Similarly, a meshwork of randomly arranged loosely packed granule-associated α-syn filaments, detected in the cytoplasm of neurons, represents the main component of NCIs (Arima et al. 1992). The distribution of NCIs seems to be unrelated to GCI load and independent of the clinical phenotype, rather reflecting regional susceptibility and increasing in density with disease duration (Cykowski et al. 2015).

NNIs are composed of densely arranged fibrils often organized in bundles, which are located beneath the nuclear membrane (Nishie et al. 2004). They may coexist with NCIs, their distribution is unrelated to GCI density and increases with disease duration. GCIs are highly concentrated within the pontine nuclei and it has been postulated that NCI formation may represent an early event during MSA pathogenesis (Nishie et al. 2004).

### Distribution of lesions and clinical correlates

GCI deposition and neurodegeneration are widespread phenomena in MSA, involving both the white and the gray matter and reflecting the multisystem nature of the disease. Nonetheless, neurodegenerative changes occur in an anatomically selective manner with the striatonigral (SND) and the olivopontocerebellar (OPCA) structures being most affected. According to a large neuropathological European cohort, SND and OPCA involvement was documented in up to 34% and 17% of MSA patients respectively, with 49% of patients showing concomitant degeneration of both systems (Ozawa et al. 2004). In Japanese natives, SND was found in 18% and OPCA in 40% of MSA patients, with 42% of patients showing SND and OPCA co-pathology (Ozawa et al. 2010; Hwang et al. 2019). The burden of neurodegenerative changes observed in each of the two systems typically reflects the predominant clinical phenotype.

Despite a low density of GCIs is typically observed in the SN, neurodegenerative changes affecting this area are marked, suggesting that local vulnerability factors may contribute to neurodegeneration (Ozawa et al. 2004; Ahmed et al. 2012). Within the striatonigral system, the dorsolateral caudal putamen and the caudate nucleus are typically the most affected areas (Kume et al. 1993; Sato et al. 2007). The dorsolateral region of the SN pars compacta (SNc), the globus pallidus and the subtalamic nucleus are also involved. As a functional counterpart, a disruption of both direct and indirect basal ganglia outflow pathways occurs (Churchyard et al. 1993; Ito et al. 1996). Clinically, akinesia has been shown to correlate with putaminal and nigral degeneration, while tremor did not correlate with cell loss in any area (Wenning et al. 1997). The widespread loss of neurons expressing both D1- and D2-like dopamine receptors justifies the L-Dopa unresponsiveness, which is typically seen at established MSA stages (Churchyard et al. 1993; Ito et al. 1996).

The Purkinje cells of cerebellar vermis and hemispheres, the inferior olivary, the dentate nuclei and the pontine basis are the most affected structures in OPCA, whose involvement correlates clinically with cerebellar ataxia. A marked loss of pontocerebellar fibers of the middle cerebellar peduncle is also seen and is thought to occur because of a “dying back mechanism” (Wenning et al. 1996b).

Pyramidal signs in MSA patients (e.g., Babinski sign, brisk reflexes) have been reported in approximately 54% of patients (Geser et al. 2006) and have been shown to correlate with neurodegenerative changes of the pyramidal tract (Wenning et al. 1997). The motor and premotor cortex show a significant burden of GCIs and neurodegeneration and myelin pallor are also reported (Tsuchiya et al. 2000; Su et al. 2001). The neurodegenerative changes mostly affect Betz cells within the lamina V of the motor cortex and are thought to be secondary to the striatonigral dysfunction (Brenneis et al. 2007).

The olfactory bulb is relatively spared in MSA compared to PD, likely accounting for a more common preservation of olfaction in patients with MSA (Kovács et al. 2003).

Various brainstem nuclei are also affected. The involvement of several brainstem areas accounts, at least in part, for MSA’s multidomain autonomic dysfunction, to which both central (supraspinal and spinal), as well as peripheral mechanisms contribute. Involved nuclei include the dorsal motor vagus nucleus and the nucleus ambiguus (cholinergic nucleus), which participate in the regulation of digestive and cardiovagal functions, and the Edinger-Westphal nucleus, a parasympathetic structure modulating pupillary reflexes (Jellinger 2014b). The loss of C1-catecholaminergic neurons in ventrolateral medullary nuclei is also reported and is thought to account, at least in part, for the development of severe cardiovascular autonomic failure in the disease (Benarroch et al. 1998). Neurodegenerative changes also occur in the hypothalamus, especially the periventricular and suprachiasmatic nuclei, potentially accounting for circadian rhythm disruption and impaired cardiocirculatory function (Ozawa 2007). The ponto-medullary reticular formation with the serotoninergic nucleus raphe and the noradrenergic locus coeruleus (LC) are also affected by neurodegenerative changes (Ozawa 2007; Jellinger 2014a). The degeneration of these areas may contribute to a broad constellation of symptoms including sleep disorders, respiratory and cardiovascular dysfunction, as well as mood disorders (Ozawa 2007).

The spinal cord is also affected in MSA. The degeneration of preganglionic cholinergic neurons of the intermediolateral column of the thoracic spinal cord is considered the main determinant of neurogenic orthostatic hypotension, while the neuronal loss in the lumbar tract of the intermediolateral column is thought to account for sphincter-detrusor dyssynergy causing urinary dysfunction in MSA (Wenning et al. 1997). Degeneration of the Onuf’s nucleus, a small structure located in the ventral horn of the sacral spinal cord, may furthermore account for urogenital dysfunction in MSA (Jellinger 2018a). Neurodegenerative changes affecting the anterior horns and the lateral corticospinal tracts of patients with MSA-c may contribute to myoclonus according to some recent observations (Hwang et al. 2019).

α-synuclein inclusion pathology can be found in skin nerve fibers, sympathetic ganglia, Schwann cells and enteric nervous system, suggesting a concomitant involvement of the peripheral nervous system. Intriguingly, α-synuclein skin deposits mostly affect somatic fibers of subepidermal plexi, with relative sparing of the unmyelinated autonomic skin fibers, which are on the other hand most affected in patients with Parkinson’s Disease with prominent orthostatic hypotension (Donadio et al. 2020).

### Other patterns

The label “minimal change” MSA was originally used to designate a pattern of widespread GCI accumulation associated with selective degeneration in the SN and locus coeruleus (Wenning et al. 1994) Subsequently a “minimal changes” variant affecting the OPCA has been described (Wakabayashi et al. 2005). The meaning of these variants is still under discussion, but the “minimal change” pattern seems to represent a neuropathological marker of more aggressive and rapidly progressive forms, possibly due to early and severe involvement of some key brainstem areas (Wenning et al. 1994; Ling et al. 2015).

GCIs have been also detected incidentally in patients without any signs or symptoms of MSA (Parkkinen et al. 2007; Fujishiro et al. 2008; Kon et al. 2013). Whether GCIs should be considered a mere incidental finding or a marker of premotor or prodromal MSA is a matter of debate (Wakabayashi et al. 2005; Ahmed et al. 2012).

In patients with young disease onset MSA (YOMSA—e.g., disease onset before 40 years old), “minimal change” patterns were slightly more common than in patients with late onset (Batla et al. 2018), even though a combination of both OPCA and SND mostly occurred (Jellinger 2012c; Batla et al. 2018).

Rare MSA variants with long disease duration (more than 15 years) have been also described; the reported cases showed diffuse GCIs accumulation with severe striatonigral and/or olivopontocerebellar degeneration with minimal involvement of the amygdala and of the limbic system (Jellinger 2012a) and no significant LB or amyloid-ß pathology (Jellinger 2012a; Petrovic et al. 2012). As a clinical counterpart, these patients showed very late-onset autonomic failure and the occurrence of autonomic complaints marked the beginning of a more rapid disease progression (Petrovic et al. 2012).

Overt dementia is considered an exclusion criterion for the diagnosis of MSA, nevertheless cognitive impairment may occur, especially in the advanced disease stages (Eschlböck et al. 2020). Regarding cognitively impaired MSA patients, concomitant amyloid-plaques, tau and LB co-pathology have been reported (Jellinger 2020). On the other hand, an increased NCI load in the hippocampus and in limbic structures has been observed in MSA patients with memory complaints compared to those without (Miki et al. 2020). The neuropathological correlates of cognitive impairment in MSA deserve anyway further studies.

### Concomitant pathologies

Lewy Bodies (LB) have been observed in up to 5–20% of MSA brains in European and US (Wenning et al. 1995; Ozawa et al. 2004; Jellinger 2007; Miki et al. 2019; Koga et al. 2020) but not in Japanese cohorts (Ozawa et al. 2010). They are mostly detected in the SN and brainstem nuclei, more rarely in limbic structures (transitional pattern) and, occasionally, diffuse LB patterns are observed (Koga et al. 2020). Some authors suggest that MSA patients with diffuse or transitional LB co-pathology may more often develop cognitive impairment and visual hallucinations and may be misdiagnosed with Dementia with Lewy Bodies (DLB), an α-synucleinopathy in which autonomic dysfunction may be severe and occur early in the disease course, or PD (Koga et al. 2020).

Alzheimer’s Disease (AD) co-pathology was thought to be detectable more rarely in MSA compared with age-matched healthy controls (Jellinger 2007) but recent studies reported amyloid-ß co-pathology in up to 38% of MSA patients (Robinson et al. 2018). Although a role of such a co-pathology in promoting cognitive impairment in MSA has been hypothesized, the clinical counterpart of these findings needs to be explored.

Tau co-pathology has also been rarely reported, with tau aggregates mostly localized in glial cytoplasm and not co-localizing with GCIs (Nagaishi et al. 2011; Jellinger 2012b). Recently an association between 3R tau glial inclusions and prolonged disease survival has been postulated (Homma et al. 2020).

TAR DNA-binding protein of 43 kDa (TDP-43) related pathology is rare in MSA and mostly occurs in the medial temporal lobe (Geser et al. 2006; Koga et al. 2018). Similarly, FUS-related pathology is typically not detected in MSA (Geser et al. 2006).

### Staging system

**Fig. 1:**
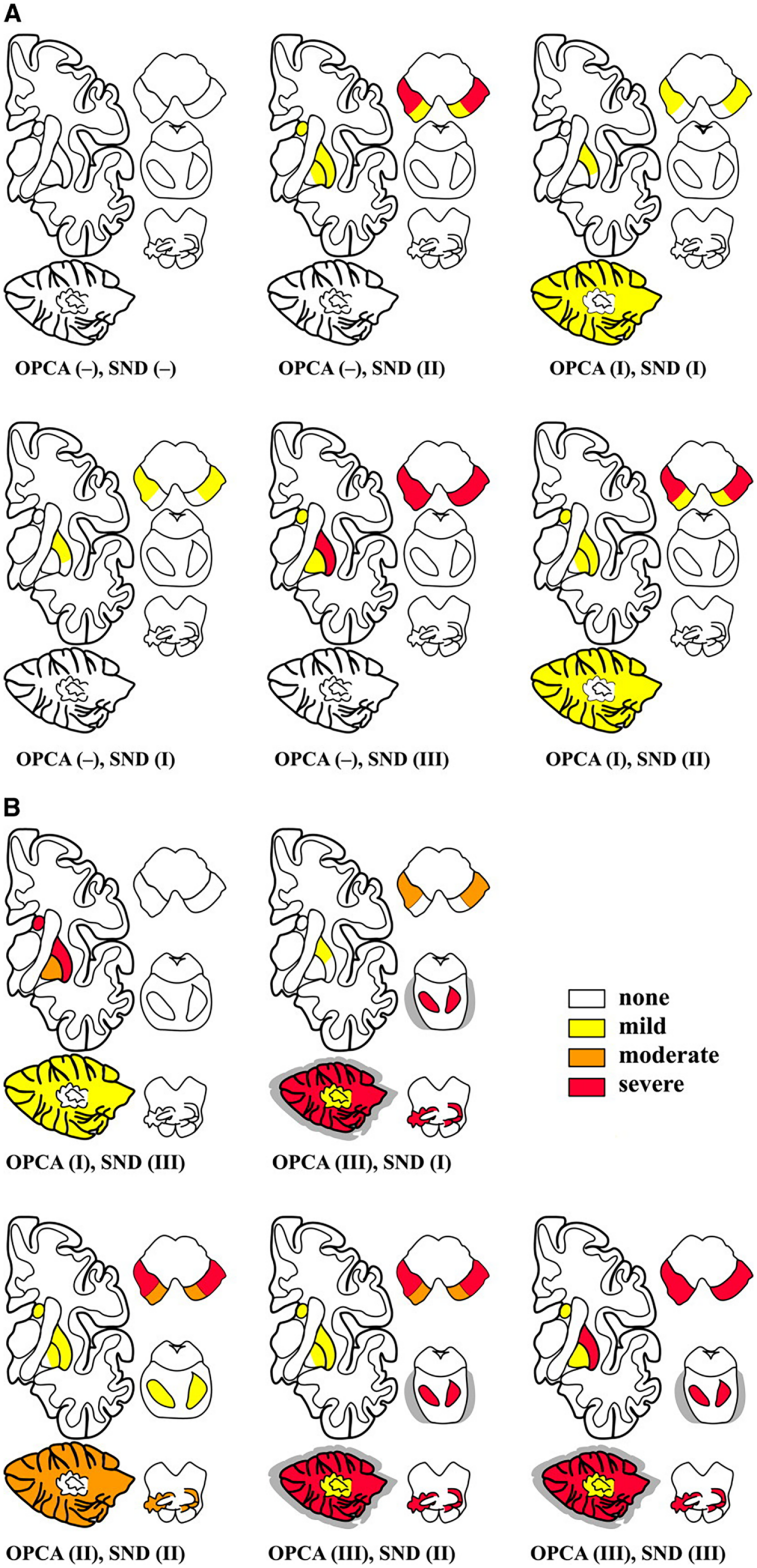
The MSA neuropathological scoring system (Reproduced from Jellinger K. et al., Mov Disord 2005, with the permission from John Wiley and Sons Ltd)

Aiming at systemizing the assessment of the MSA neuropathological spectrum, a scoring system has been proposed in 2005 by Jellinger and colleagues (Wenning et al. 2002; Jellinger et al. 2005) (Fig. 1). Based on a semiquantitative assessment of four main features, namely GCI load, atrophy, gliosis and neuronal loss, a framework for the standardized assessment of SND has been developed. The four abovementioned neuropathological hallmarks should be assessed in 8 anatomical regions: putamen (ventromedial ad dorsolateral parts), caudate nucleus (anterior and posterior regions), globus pallidus (medial and lateral parts) and SNc (mediobasal and dorsolateral regions). Each feature is rated as follows: absent (0), minimal ( ±), slight ( +), moderate (+ +), and severe (+ + +).

Accordingly, three grades of severity have been defined for SND:

- SND grade I: degeneration restricted to the SN, also referred as “minimal changes” pattern.
- SND grade II: degeneration involving the SN and mild involvement of the putamen.
- SND grade III: both the SN and putamen are severely involved, accompanied by mild involvement of caudate and globus pallidus.
  An analogous scoring system has been applied to OPCA, where four regions have been evaluated, namely the pontine nuclei, the cerebellum, the inferior olive and SNc (Jellinger et al. 2005), thus identifying:
- OPCA grade I: diffuse GCIs pathology associated with mild loss of Purkinje cells and myelin pallor in the cerebellum; mild to severe cell loss in the SNc is often also found.
- OPCA grade II: minimal cerebellar atrophy with mild Purkinje cell loss and demyelination, mild gliosis and neuronal loss in the pontine and inferior olives.
- OPCA grade III: severe cell loss in the cerebellum (both vermis and hemispheres), the pontine nuclei and inferior olives.

The combination of different grades of SND and OPCA enables the identification of several neuropathological patterns.

Similarly, the subsequent involvement of different brain areas in the neurodegenerative changes occurring in MSA-p and MSA-c has been highlighted by Halliday and colleagues in 2011 (Halliday et al. 2011).

The Braak&Braak staging system well reproduces the progression of α-synuclein accumulation and related neurodegeneration in LB-related disorders (Braak et al. 2003) but fails in explaining the clinical and neuropathological progression of MSA, which is a primary oligodendroglioneural pathology (Jellinger 2018a). The neuropathological scoring systems developed for MSA well correlate with the initial clinical presentation and with disease severity, but they do not take into account the progressive involvement of key autonomic nuclei and are not able to predict the disease progression and the spreading of the α-synuclein pathology.

## From neuropathology to the understanding of pathogenesis: what we learned and what we need to learn

The etiology and pathogenesis of MSA still remains largely elusive; nonetheless since the original description of the GCIs in SND, OPCA and Shy Drager syndrome in 1989 (Papp et al. 1989), several steps forward have been made to untangle some key pathogenic events in MSA (Meissner et al. 2019). Although the clinical presentation of MSA seems to be the expression of an underlying diffuse neuronal cell loss, the strict correlation between disease severity and GCIs load, along with evidence coming from in vitro and in vivo animal models, supports the notion that MSA is a primary oligodendrogliopathy with secondary neurodegeneration (Wenning et al. 2008; Jellinger 2018a).

α-synuclein accumulation seems to be a pivotal event in inducing glial dysfunction; nonetheless, both the reasons of its selective accumulation within glial cells and the source of α-synuclein aggregating in oligodendrocytes remain poorly understood. This protein is natively expressed at low levels in glial cells and its concentration decreases with oligodendrocytes maturation (Miller et al. 2005; Djelloul et al. 2015). The uptake of oligomeric α-synuclein forms from the surrounding environment has been postulated and, accordingly, an intense cell-to-cell transfer, involving both neuron-to-neuron and neuron-to-glia crosstalk mechanisms, has been shown in vitro (Desplats et al. 2009; Woerman et al. 2015; Cavaliere et al. 2017) and in vivo (Reyes et al. 2014). Moreover, a self-templating propagation mechanism resembling prion´s seeding activity has been observed, leading to the notion of a connectome-dependent prion-like propagation mechanism for α-synuclein (Luk et al. 2012; Watts et al. 2013; Prusiner et al. 2015; Woerman et al. 2015; Cavaliere et al. 2017). Nonetheless, lack of evidence for infectivity and inter-human transmission differentiates MSA from prion diseases (Heras-Garvin and Wenning 2016; Heras-Garvin and Stefanova 2020b). Mechanisms leading to selective glial dystrophic accumulation of α-synuclein and to the formation of GCIs rather than LBs still remain elusive. Nonetheless, the existence of different α-synuclein structural conformers, also referred to as strains, with different features and behavior has been hypothesized (Desplats et al. 2009; Fellner et al. 2020). This notion is supported by several experimental observations, suggesting that different synthetic α-synuclein conformers show distinct toxicity, seeding potential, tau cross-seeding activity both in vitro and in vivo after local and systemic administration in animal models (Bousset et al. 2013; Guo et al. 2013; Peelaerts et al. 2015; Rey et al. 2019; Lau et al. 2020). Besides recombinant synthetic strains, brain-derived α-synuclein aggregates obtained from MSA, PD and DLB patients also exhibit distinct seeding activity, propagation patterns and toxicity both when applied to cultured cells (Woerman et al. 2015; Yamasaki et al. 2019; van der Perren et al. 2020) and to animal models (Watts et al. 2013; Prusiner et al. 2015; Rey et al. 2019; van der Perren et al. 2020). Accordingly, distinct GCI and LB strains are supposed to account, at least in part, for the different clinical phenotype and progression of α-synucleinopathies (Prusiner et al. 2015; van der Perren et al. 2020). The recent characterization of ultrastructural differences between α-synuclein filaments detected in GCIs and in Lewy Bodies supports this hypothesis and may represent the biochemical substrate of the distinct strains’ behaviors (Schweighauser et al. 2020).

Further in vivo insights of a selective strain-specific seeding and self-templating activity, differentiating Lewy Bodies- from GCI-derived strains, are provided by protein seeding amplification techniques like the protein misfolding cyclic amplification assay (PMCA) (Shahnawaz et al. 2020). According to some preliminary results, these techniques could be able to differentiate in vivo MSA- from PD-α -synuclein strains when assessed in the cerebrospinal fluid (CSF) of affected subjects, bearing a great potential for the development of MSA fluid biomarkers and for improving the early and differential diagnosis across α-synucleinopathies (Fellner et al. 2020; Shahnawaz et al. 2020). The exact mechanisms underlying the formation of distinct strains are not clarified, but glial-specific features of the intracellular milieu are thought to promote the selective α-synuclein misfolding and accumulation within glial cells and partially justify the different behavior of α-synuclein in MSA compared to other α-synucleinopathies (Peng et al. 2018). The relocation of phosphoprotein-25α (p25α) from myelin to glial cytoplasm and nucleus, which is thought to be an early event in MSA, seems to promote α-synuclein aggregation and misfolding thus enhancing the deposition of GCIs (Song et al. 2007; Ota et al. 2014; Mavroeidi et al. 2019). Other pathophysiological mechanisms potentially contributing to neurodegeneration have been described including mitochondrial dysfunction, alteration in cell-death regulatory mechanisms and oxidative stress (Jellinger 2014a; Heras-Garvin and Stefanova 2020a). By inducing microglial activation, α-synuclein may contribute to neuroinflammation, thus exacerbating and accelerating the neurodegeneration (Heras-Garvin and Stefanova 2020b).

Key pathophysiological events in MSA can be explored and recapitulated by means of in vitro and in vivo models. These need to reflect both the main clinical features of MSA and its neuropathological hallmarks (e.g., GCIs accumulation). To date three main approaches have been used to develop animal models of MSA (Table 1) based, respectively, on the use of neurotoxins selectively damaging the nigrostriatal or olivopontocerebellar structures, recombinant genetic techniques and viral vectors aiming at inducing α-synuclein overexpression in oligodendrocytes (Jellinger 2019b; Lee et al. 2019). A further in vitro model of MSA based on the use of induced pluripotent stem cells has been recently proposed (Abati et al. 2018; Monzio Compagnoni et al. 2018; Nakamoto et al. 2018; Ndayisaba et al. 2019; Herrera-Vaquero et al. 2021). Moreover, models based on the inoculation of MSA patients’ brain-derived homogenates in α-synuclein overexpressing mice have been shown to reproduce the α-synuclein prion-like spreading pattern. Nevertheless, a transgenic background is required to promote α-synuclein spreading and no GCIs are observed in these animals (Watts et al. 2013; Prusiner et al. 2015; Ding et al. 2020; van der Perren et al. 2020). The translatability from bench to bedside of each of the abovementioned models needs to be broadly assessed and further technological advances improving the post-mortem exploration of the human neuropathology offer the opportunity to explore their human counterpart.

**Table 1:** Overview of in vitro and in vivo models of MSA

In vitro models				
Model	Cell line	Mechanism	Features	
U-373 MG (Stefanova et al. 2005)	Human glioblastoma astrocytoma cells	Overexpression of α-synuclein	Increased susceptibility to oxidative stress and neuroinflammation	
OLN-93 (Kragh et al. 2009)	Rat oligodendrocytes	Overexpression of α-synuclein and p25α	Impaired microtubules remodeling and promoted apoptosis	
CG4 (May et al. 2014)	Rodent oligodendrocytes	Overexpression of α-synuclein	Impaired cellular maturation due to the inhibition of MBP expression	
iPSCs (Nakamoto et al. 2018)	Human-induced pluripotent stem cells	In vitro differentiation	Mitochondrial deficits	
iPSCs (Monzio Compagnoni et al. 2018)	Human-induced pluripotent stem cells	In vitro differentiation	Mitochondrial deficits	
iPSCs (Herrera-Vaquero et al. 2021)	Human-induced pluripotent stem cells	In vitro differentiation	Mitochondrial deficits, increased susceptibility to oxidative stress	

In vivo models				
Model	Specie(s)	Neuropathology	Clinical phenotype	Features
Neurotoxin-based				
6-OHDA + QA (Wenning et al. 1996a)	Rat (intrastriatal)	Striatonigral degeneration (without GCIs)	Poorly L-Dopa responsive motor impairment (akinesia, rigidity); no autonomic features	GCIs pathology not reproduced Lesions fail to spread outside basal ganglia No autonomic impairment
MPTP + 3-NP (Ghorayeb et al. 2000; Stefanova et al. 2003)	Mouse, non-human primate (intrastriatal, systemic – e.g., i.v., i.p.)	Striatonigral degeneration (without GCIs)	Poorly L-Dopa responsive motor impairment (akinesia, rigidity); no autonomic features	GCIs pathology not reproduced Lesions fail to spread outside basal ganglia No autonomic impairment
Transgenic α-synuclein overexpression models				
PLP α-syn (Kahle et al. 2002)	Mouse	Striatonigral degeneration (with GCIs), loss of neurons in central autonomic centers of CNS and spinal cord; OPCA appears when triggered by treatment with 3-NP; GCIs-related microglial activation	Autonomic dysfunction predating motor deficits (exacerbated by treatment with 3-NP). Intact olfaction; sleep structure disruption resembling RBD	Both autonomic and motor dysfunction are replicated; Only model showing GCI-related microglial activation; The mild motor phenotype represents the main limitation
CNP α-syn (Yazawa et al. 2005)	Mouse	Striatonigral, cerebellar, callosal and cortical demyelination and axonal degeneration (with α-synuclein glial inclusions); severe astrogliosis	Motor deficits (exacerbated by treatment with 3-NP)	Pathological substrate of motor deterioration seems to be different from SND and OPCA; Useful to assess protein interactions
MBP α-syn (Shults et al. 2005)	Mouse	Disrupted axonal integrity in striatum, brainstem, cerebellum without neuronal loss in SN (with α-synuclein glial inclusions); mild demyelination and astrocytosis of white matter tracts	Motor deficits. High expressor line 29 shows reduced survival, mild expressor line 1 shows preserved survival but with mild motor phenotype	Pathological substrate differs from human MSA (e.g., no SN neuronal loss); No microglial activation; Useful in recapitulating key pathogenetic mechanisms
Viral-mediated α-synuclein overexpression models				
AAV Olg001 (Mandel et al. 2017)	Non-human primate	Demyelination of white matter tracts and corpus callosum, microgliosis in the striatum (with widespread α-synuclein glial inclusions)	Needs to be better assessed	Recapitulates some main neuropathological and clinical features of MSA but deserves further validation
AAV1/2 MBP promotor (Bassil et al. 2017)	Rat, non-human primate (macaque)	Dopaminergic cell loss (with widespread α-synuclein glial inclusions)	L-Dopa unresponsive progressive motor deficits	Recapitulates some main neuropathological and clinical features of MSA but deserves further validation
α-synuclein spreading models				
Tg83 ± mouse inoculated with MSA-derived α-synuclein (intracerebral and peripheral inoculation) (Watts et al. 2013; Prusiner et al. 2015; Ding et al. 2020)	Mouse	Widespread α-synuclein inclusion pathology, no GCIs	Various degrees of motor impairment and autonomic dysfunction	Recapitulate the prion-like spreading of MSA-derived α-synuclein; Require a transgenic background or viral-mediated α-synuclein overexpression (no inclusion pathology in wild-type mice) No GCIs
Tg(SNCA*A53T + / +)Nbm mouse inoculated with MSA-derived α-synuclein (Woerman et al. 2019)				
rAAV2/7 A53T α-synuclein overexpressing mouse inoculated with MSA-derived α-synuclein (van der Perren et al. 2020)				

*3-NP* 3-nitropropionic acid, 6-OHDA: 6-hydroxydopamine, *AAV* adeno-associated virus, *CG4* central glia 4, *CNP* cyclic nucleotide 3’-phosphodiesterase, *GCI* glial cytoplasmic inclusion*,* *iPSC* induced pluripotent stem cells, *i.p.* intra-peritoneal, *i.v.* intra-venous, *MBP* myelin basic protein, *MPTP* 1-methyl-4-phenyl-1,2,3,6-tetrahydropyridine, *MSA* multiple system atrophy*,* *OPCA* olivopontocerebellar atrophy, *PBMC* peripheral blood monocyte cells, *PLP* proteolipid protein, *QA* quinolinic acid, *RBD* REM sleep behavior disorder, *SND* striatonigral degeneration

Until now all neuroprotection clinical trials in MSA, with the exception of some positive preliminary results with mesenchymal stem cells, have failed in reaching their primary outcomes (Meissner et al. 2019). Currently, most experimental drugs investigated in preclinical and clinical settings aim at interfering with α-synuclein misfolding and accumulation, both by means of α-synuclein aggregation inhibitors, α-synuclein clearing enhancers and immunotherapy approaches. The modulation of neuroinflammation and of microglial activation are also regarded as promising strategies (Heras-Garvin and Stefanova 2020a).

## Jellinger’s contribution in unravelling the nature of the beast

Since the first clinical reports of MSA dating back to the beginning of the nineteenth century and the unification of three main clinical phenotypes, namely the striatonigral degeneration, the olivopontocerebellar atrophy and the Shy Drager syndrome, under the unique label of MSA by Graham and Oppenheimer in 1969 (Graham and Oppenheimer 1969), several steps forward have been made in the understanding of the main clinical features, neuropathological hallmarks and, at least in part, pathophysiological events underlying this multifaceted disorder. In this frame, we would like here to honor the seminal contribution of the Austrian neurologist and neuropathologist Kurt Jellinger in unravelling some key neuropathological aspects of this disease.

Jellinger’s contribution to the understanding of pathological hallmarks of neurodegenerative disorders like Parkinson’s (PD) and AD is substantial and world-renowned; his contribution in the field of an orphan disease like MSA has also been substantial.

Worldwide recognized for the development of the first neuropathological grading system for MSA (Jellinger et al. 2005), Kurt Jellinger not only contributed to the development of a standardized semi-quantitative method for the assessment of the underlying neuropathology severity but also tried to assess its value in explaining the observed clinical phenotypes. Jellinger further explored the neuropathological correlates of clinical endophenotypes, assessing, for example, the pathological distinctive features of cognitive impairment in MSA (Jellinger 2020), of YOMSA (Jellinger 2012c), old-onset (Jellinger 2018b) and long-disease duration MSA (Jellinger 2012a). Stepping aside from the traditional classification of proteinopathies, he explored the prevalence and meaning of concomitant pathologies in MSA, contributing to overcome the traditional classification of this disorder as a pure α-synucleinopathy. He also contributed to the development of MSA animal models (Scherfler et al. 2000).

Besides every single original contribution, the constant strive for finding in the histological sections answers to burning clinical questions probably represents Jellinger’s most important contribution to the field and, broadly speaking, to our understanding of neurodegenerative disorders.

**Fig. 2:**
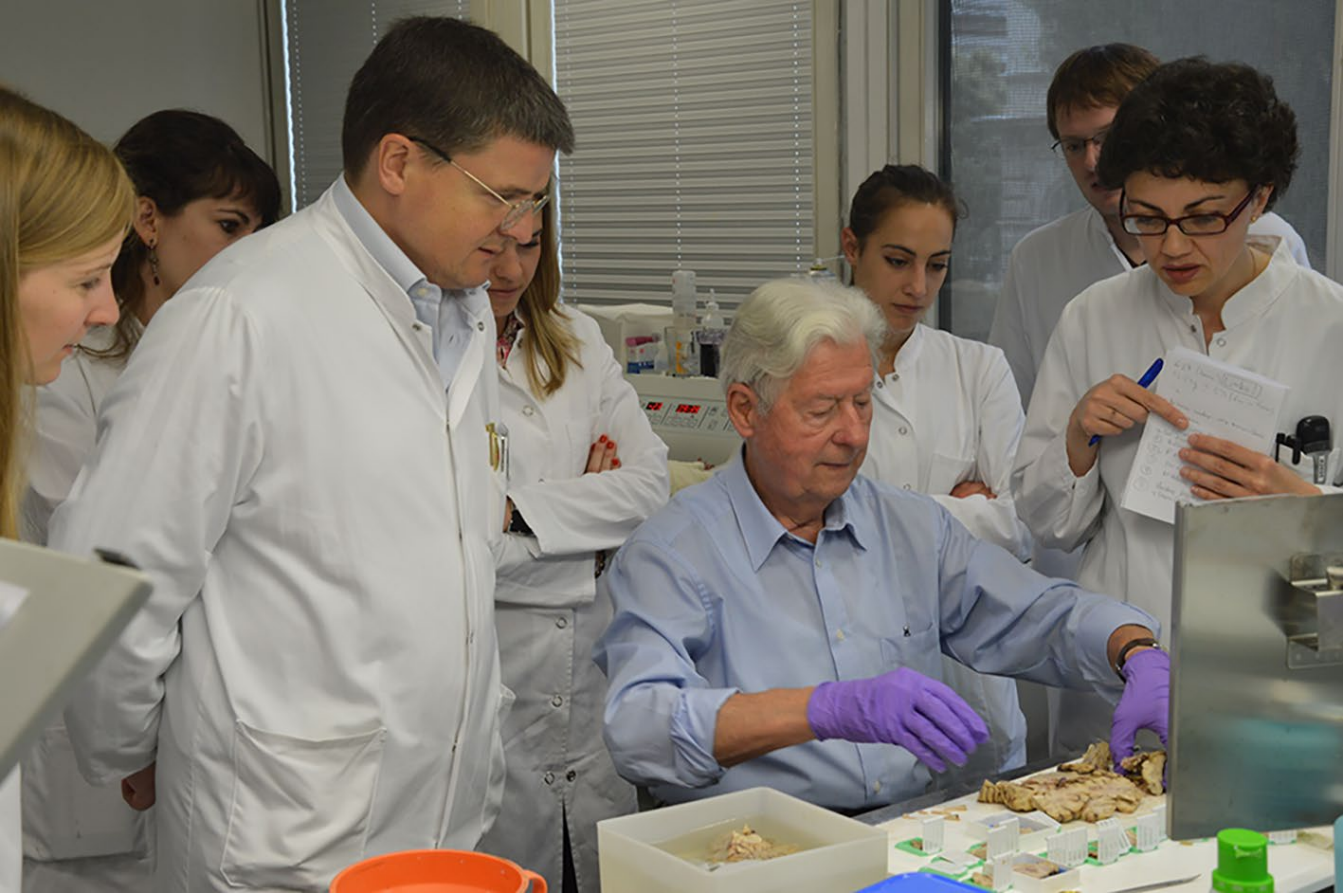
Prof. Kurt Jellinger with the team of the Innsbruck Division of Neurobiology: an enthusiastic teacher motivating young scientists

Although much has been achieved, much should still be done to unravel the elusive nature of MSA and to improve tailored models and disease-modifying approaches. In this context, Jellinger’s curiosity and ability to work both at the bench and at the bedside represent an inspiring example for both young neurologists and neuropathologists (Fig. 2).
