# Correction to: Neuropathology of multiple system atrophy: Kurt Jellinger`s legacy

**DOI:** 10.1007/s00702-021-02412-1

**Published:** 2021-09-08

**Authors:** Nicole Campese, Alessandra Fanciulli, Nadia Stefanova, Johannes Haybaeck, Stefan Kiechl, Gregor K. Wenning

**Affiliations:** 1 Neurology Unit, Department of Clinical and Experimental Medicine, University of Pisa, Via Roma 67, 56126 Pisa, Italy; 2 Department of Neurology, Medical University of Innsbruck, Anichstrasse 35, 6020 Innsbruck, Austria; 3 Institute of Pathology, Neuropathology and Molecular Pathology, Medical University of Innsbruck, Müllerstrasse 44, 6020 Innsbruck, Austria; 4 Diagnostic & Research Center for Molecular BioMedicine, Institute of Pathology, Medical University Graz, Neue Stiftingtalstrasse 6, 8010 Graz, Austria

## Correction to: Journal of Neural Transmission 10.1007/s00702-021-02383-3

In the original version of this article there was a mistake in reporting some references. The correct information is given below.

In section “Dissection of lesions and clinical correlates”, in the fourth sentence of the first paragraph, the reference “Hwang et al. 2019” is not pertinent. The correct sentence should read:

In Japanese natives, SND was found in 18% and OPCA in 40% of MSA patients, with 42% of patients showing SND and OPCA co-pathology (Ozawa et al. 2010).

In section “Concomitant pathologies”, fourth paragraph should read:

TAR DNA-binding protein of 43 kDa (TDP-43) related pathology is rare in MSA and mostly occurs in the medial temporal lobe (Geser et al. 2011; Koga et al. 2018). Similarly, FUS-related pathology is typically not detected in MSA (Geser et al. 2011).

The authors apologize for this errors, which do not impact on the scientific content of the paper.
